# Production and Enhancement of Omega-3 Fatty Acid from *Mortierella alpina* CFR-GV15: Its Food and Therapeutic Application

**DOI:** 10.1155/2014/657414

**Published:** 2014-05-21

**Authors:** Ganesan Vadivelan, Govindarajulu Venkateswaran

**Affiliations:** Food Microbiology Department, Central Food Technological Research Institute, Mysore, Karnataka 570020, India

## Abstract

*Mortierella* sp. has been known to produce polyunsaturated fatty acids (PUFAs) such as GLA and AA under normal growth medium conditions. Similarly, under the stress condition, this fungus produces EPA and DHA in their mycelial biomass. Among the 67 soil samples screened from the Western Ghats of India, 11 *Mortierella* isolates showed the presence of omega-6 and omega-3 fatty acid, mainly GLA, AA, EPA, and DHA in starch, yeast-extract medium. Nile red and TTC strains were used for screening their qualitative oleaginesity. Among the representative isolates, when *Mortierella* sp. is grown in a fat-producing basal medium, a maximum lipid content of 42.0 ± 1.32% in its mycelia, 6.72 ± 0.5% EPA, and 4.09 ± 0.1% DHA was obtained. To understand the *Mortierella* sp. CFR-GV15, to the species level, its morphology was seen under the light microscope and scanning electron microscope, respectively. These microscopic observations showed that isolate *Mortierella* sp. CFR-GV15 produced coenocytic hyphae. Later on, its 18S rRNA and the internal transcribed spacer (ITS) sequences were cloned, sequenced, and analyzed phylogenetically to 18S rRNA and ITS1 and ITS4 sequences of related fungi. This newly isolated *Mortierella alpina* CFR-GV15 was found to be promising culture for the development of an economical method for commercial production of omega-3 fatty acid for food and therapeutical application.

## 1. Introduction 

The pathogenesis of lifestyle-related diseases such as obesity, hyperlipidemia, arteriosclerosis, diabetes mellitus, and hypertension is complicated and the precise mechanisms underlying their development have not yet been elucidated. However, there is now much evidence to suggest that specific fatty acids have beneficial effects on human health which could contribute to the prevention of many chronic diseases in humans [1]. In particular, polyunsaturated fatty acids (PUFAs), such as linoleic acid (18:2, n-6), *α*-linolenic acid (LNA, 18:3, n-3), and arachidonic acid (20:4, n-6), are very important for maintaining biofunctions in mammalians as essential fatty acids [2]. Polyunsaturated fatty acids (PUFAs), a group of fatty acids containing double bonds at omega-3 position, including alpha-linolenic acid (ALA), eicosapentaenoic acid (EPA), and docosahexaenoic acid (DHA), have many specialized health benefits. PUFAs are found to be helpful in treating hypertension, Crohn's disease, rheumatoid arthritis, and asthma. Preview reported reducing the risk of primary cardiac arrest and coronary artery disease and reducing serum triglycerides. Also, encouraging data supports a role of omega-3 fatty acids in the prevention of breast and lung cancer [3]. Recent evidence suggests that omega-3 fatty acids may also have beneficial effects on mood disorders, including major depression and bipolar disorder, schizophrenia, and dementia, and reduce serum triglycerides [4].

As these essential fatty acids cannot be accumulated and synthesized in the human body, they must be derived from dietary sources. Current dietary sources of omega-3 PUFAs (EPA and DHA) from animals include fatty fish species, such as Herring, Mackerel, Sardine, and Salmon [5, 6]. The quality of the fish oil, however, is changing and depends on the type of fish, seasonal time, and place of fishing. The applications of fish oil in foods, infant formulas, or pharmaceuticals have some disadvantages because of their contamination by environmental pollution such as heavy metal accumulation and regular fishy smell and unpleasant taste. Moreover, the majority of the PUFAs in their body emerge from their food though they had some capacity for de novo biosynthesis of omega-3 fatty acids. The plant sources include green leaves and a variety of vegetable oils like flax, hemp, rapeseed (canola), soybean, and walnut, which are quite rich in alpha-linolenic acid (ALA). The ALA is the “parent” fatty acid of EPA and DHA and human body converts ALA rapidly into EPA and more slowly into DHA [7]. However, since the production building and downstream processing of oils from plants and fishes are cost-effective and time-consuming, it is likely that methods for the microbial production of oils are more convenient and should be focused on in the near future. Although marine algae and fungi are used in commercial production, the difficulties in cultivation and adaptation of marine microorganisms have changed the situation from conventional marine sources into nonmarine microorganisms. One such fungus,* Mortierella*, a member of the family* Mortierellaceae*, is the most promising microorganism for the industrial production of omega-6 or omega-3 PUFAs. Major members of the genus include* M. alliacea, M. alpina, M. polycephala, M. elongata, *and* M. spinosa*. One of the* M. alpina* has gained importance due to its higher production of lipids. Apart from being the major producer of arachidonic acid,* M. alpina* also produces other PUFAs such as linolenic acid (LA), gamma-linolenic acid (GLA), and dihomogamma-linolenic acid (DGLA); EPA and DHA, in fewer amounts, [8, 9] suggest that this fungus is a potential producer of many biologically important PUFAs, both omega-3 and omega-6. More enhancement of omega-3 fatty acid production by* M. alpina* would be significant since they are playing a major role in brain development and the prevention of depression-related disorders. Hence the physiology of* M. alpina* can be appropriately altered by several means, for example, induced mutation, chemical inhibitors, stress conditions, cultural changes, and so on, in order to enhance the biosynthesis of omega-3 fatty acids (EPA and DHA) instead of omega-6 fatty acids (GLA and AA). We report in this paper the production of omega-3 fatty acids by* Mortierella alpina* CFR-GV15 by specific cultural methods and their application in food and therapeutic purpose.

## 2. Material and Method

### 2.1. Soil Sampling

Collection of saprophytic soil samples from various locations in South India, mostly from Tamil Nadu, Kerala, and Karnataka of the Western Ghats area, was performed. Selectively untouched soil samples were collected from the bottom of 5 to 10 cm, sealed in sterile sampling polythene bags, and brought to the laboratory for further analysis.

### 2.2. Screening and Isolation of* Mortierella *sp.

The soil samples were screened by spread plate techniques by the following method. 10.0 gm of soil sample was suspended in 90 mL sterile saline water, serial 10-fold dilutions of that suspension were made [10], and then 0.1 mL of saline water from the 10^−2^ dilution was spread evenly on the surface of MEA (malt extract agar) medium. The plate was then incubated at 6°C in a cellular refrigerator. After 8–12 days of incubation, the selective fungal white colonies were transferred to fresh potato dextrose agar and incubated at room temperature (28°C ± 1) for further purification. Then the absolute cultures were identified, maintained on the PDA plate or slants at 4°C, and subcultured once every 2 months.

### 2.3. Cultural Method and Cultivation

*M. alpina* cultures were maintained on potato dextrose agar slants at 4°C and subcultured every 2 months [11]. The seed culture was prepared in 50 mL medium containing (g/L) glucose, 20; yeast extract, 10; the pH was adjusted to 6.0 and cultures were incubated for 48 h at 28°C. The fermentation medium composition was as follows (g/L): starch, 20 g; yeast extract, 5 g; KNO_3_, 10 g; KH_2_PO_4_, 1 g; and MgSO_4_, 0.5 g. The final pH was adjusted to 6.5 and sterilized at 121°C for 15 min. The culture medium was then incubated for 7 days at 20°C temperature on a rotary shaker at 230 rpm.

### 2.4. Dry Biomass Determination and Lipid Extraction

Harvest and extraction of lipids from biomass were performed according to the procedure of Nisha et al. [11]. Biomass production was determined by harvesting the cells by suction filtration followed by drying at 55–60°C overnight. The dry biomass was ground to fine powder and packed into a thimble, macerated with 0.1 N HCl for 20 min. 1 g of fungal dry powder was blended with 40 mL of chloroform/methanol (2 : 1), the mixtures were agitated for 20 min in an orbital shaker at 20°C and then filtered with Whatman paper number 1, and 0.9% sodium chloride solution was added. The chloroform solvent containing total fatty acid then evaporated and dried under N_2_ vacuum.

### 2.5. Analytical Method 

#### 2.5.1. Methyl Ester Preparation and Fatty Acid Analysis

Fatty acid methyl esters (FAME) were prepared as described by Nisha and Venkateswaran [12] and were used for gas chromatographic analysis. FAME was prepared by using methanolic HCl in the ratio of 95 : 5 as the methylating agent and 14% BF_3_. The derivatized lipids in the hexane layer were evaporated under N_2_ and dissolved in 1 mL of benzene, and any solids were removed by centrifuging at 10,000 (rpm) for 2 min. Lipids were analyzed by gas chromatography (Shimadzu 2010 system, Japan) using RTX-2330 (fused silica) 30 m capillary column of 0.25 *μ*m internal diameter and df (*μ*m) 0.20 *μ*m. The column was operated at an initial temperature of 160°C–250°C at the rate of 5°C/min and was to hold for 10 min. The injector and detector temperature were 240°C and 250°C, respectively. Carrier gas (nitrogen) was supplied at a total flow rate of 50 mL/min with a split ratio of 20 : 0 and fatty acids were identified by comparison with standards (Sigma).

### 2.6. Triphenyltetrazolium Chloride (TTC) Staining

The two-day fresh mycelium of* Mortierella* fungus was harvested by suction filtration, and the staining procedure was done according to Zhu et al.'s [10] method with slight modification; 1 mL of 0.6% triphenyltetrazolium chloride (TTC) solution in 0.5 mol-1 phosphate buffer (pH 7.8) was added to 0.1 g of fresh mycelia in a scrub cap tube and incubated in the dark for one hour at room temperature. Mycelia were then rinsed twice with sterile water and homogenized by mortal postal grinding. Then the red triphenylformazan (TF) formed in mycelia was extracted three times with 2 mL of ethyl acetate at room temperature using fresh solvent each time. The staining level was quantified by measuring absorbance of TF in the ethyl acetate solvent at 485 nm wavelengths.

### 2.7. Genomic DNA Extraction, Sequencing, and Data Analysis

The total genomic DNA extraction was performed according to the protocol followed by Michaelson et al. [13]. The total DNA precipitation was concentrated with ethanol, dissolved, and stored in TE buffer. A DNA segment containing the 3′ end of 18S rRNA, internal transcribed spacer 1 (ITS1), 5.8S rRNA, and internal transcribed spacer 4 (ITS4) and the 5′ end of 28S rRNA was amplified by using a forward primer ITS1 (5′GGAAGTAAAAGTCGTAACAAGG) and a reverse primer ITS4 (5′TCCCCGCTTATTGATATGC) [14]. DNA amplification by PCR was performed in a total volume of 50 *μ*L. The PCR conditions were followed by the Ho et al. [9] method with slight modification by using ITS1 and ITS4 primer, with initial denaturation at 94°C for 5 min followed by 30 cycles at 94°C for 45 s, 57°C for 45 s, and 72°C for 1 min and a final extension at 72°C for 5 min. After the amplified products were separated on electrophoresis using 0.8% agarose gel and detected by staining with ethidium bromide, all PCR products were amplified from strains of* M. horticola* CFR-GV10,* M. exigus* CFR-GV11,* M. elongata* CFR-GV12,* Mortierella *sp. CFR-GV13,* Mortierella *sp. CFR-GV14,* M. alpina* CFR-GV15,* M. alpina* CFR-GVM15,* M. elongata* CFR-GV16,* M. elongata* CFR-GV17,* M. elongata* CFR-GV18, and* M. elongata* CFR GV19 and standard culture of* M. alpina* CBS 528.72 and* M. alpina* 6344 genomic DNA showed only one band. The PCR products were purified by using Qiagen and DNA Gel Band Purification Kit following the instructions of the manufacturer (Qiagen Biosciences, Germany). The final amplified PCR products were then sequenced by automatic sequencer (Chromos Biotech, Bangalore, India). The standard culture and sequences of* M. alpina* CBS 528.72,* M. alpina* MTCC 6344, recently isolated* Mortierella *sp.,* M. horticola* CFR-GV10,* M. exigus* CFR-GV11,* M. elongata* CFR-GV12,* Mortierella *sp. CFR-GV13,* Mortierella *sp. CFR-GV14,* M. alpina* CFR-GV15,* M. alpina* CFR-GVM15,* M. elongata* CFR-GV16,* M. elongata* CFR-GV17,* M. elongata* CFR-GV18, and* M. elongata* CFR GV19 were submitted to GenBank, with accession numbers KF743701, KF743702, KF743703, KF798172, KF798173, KF561137, KF683921, KF568909, KF679986, and KF679987, respectively. Standard sequence was obtained by GenBank. The sequences were aligned by using the phylogeny tree program [15], and the alignment was corrected manually. A character's matrix was calculated by the program of phylogeny tree.

### 2.8. Statistical Analysis

Data obtained from three independent analyses was expressed as mean (3SD). Experimental data was subjected to analysis of variance and Duncan's multiple range test (*P* ≤ 0.05) using the Statistical Analysis System [16].

## 3. Results and Discussion

**Figure 1:**
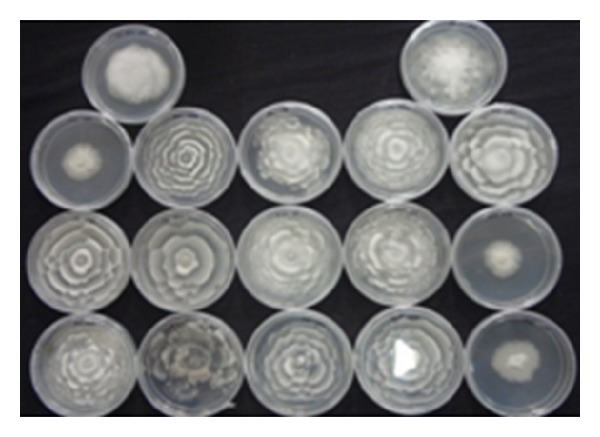
*Mortierella* sp. from soil source of roselle petal growth on PDA plate.

**Figure 2:**
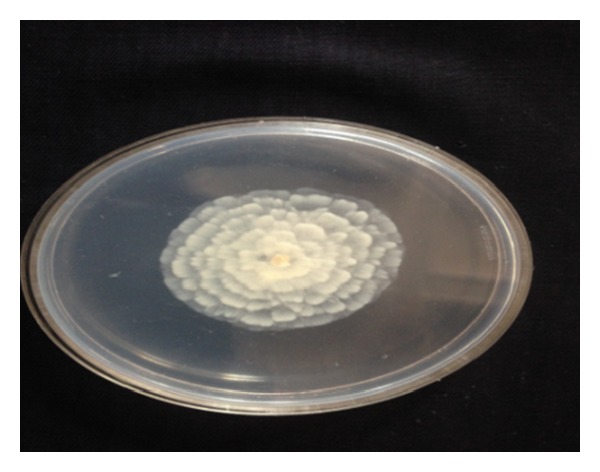
Selective isolated* M. alpina* CFR-GV15.

**Figure 3:**
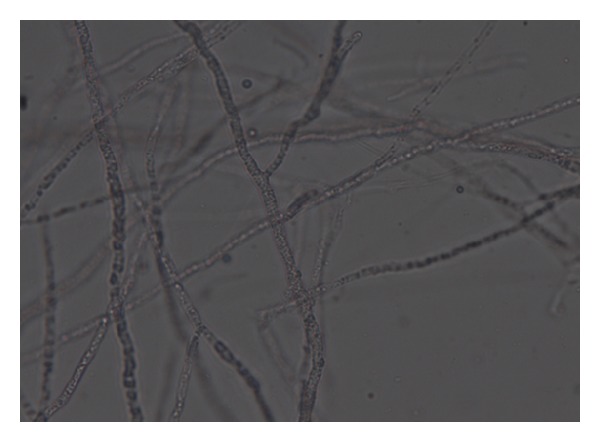
Light microscopy image of* M. alpina* CFR-GV15 mycelia.

**Figure 4:**
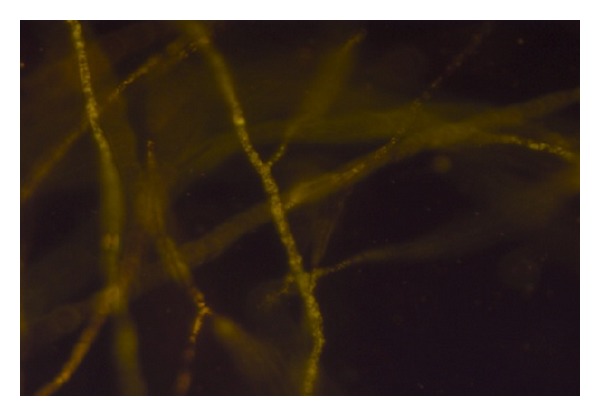
Nile red staining of* M. alpina* CFR-GV15 under fluorescence microscope.

Sixty-seven soil (Table 1) samples have been screened on MEA medium for isolation of* Mortierella* sp. for the production of omega-3 fatty acids. After the incubation period of 8–12 days at low temperature, a few white colonies were grown and these white colonies were microscopically observed for their morphological identity (Figures 1 and 2) and further confirmed with fluorescence microscope (Figures 3 and 4). Nile red staining had helped the presence of oil or lipid globules and was further observed under scanning electron microscope (SEM) for their confirmation. The morphologies of mycelial structure and sporangiophores of all the isolates were compared with the standard culture obtained from* M. alpina *MTCC 6344 and* M. alpina* CBS 528.72 cultures. Most of the features were in compliance with the standard cultures. This initial screening confirmed that most of the white colonies grown on the MEA medium were* Mortierella *sp. [17, 18]. It was also observed that all the sporangiophores were unbranched structure measuring the length between 20 and 120 *μ*m with an irregular and wide swollen base. At this stage, though all the isolated sporangiophores were very much in concomitance with* Mortierella alpina,* they were placed* Mortierella *sp. [9, 19]. Later on, 18S rRNA sequence was also compared with MTCC and CBS standard cultures.

**Figure 5:**
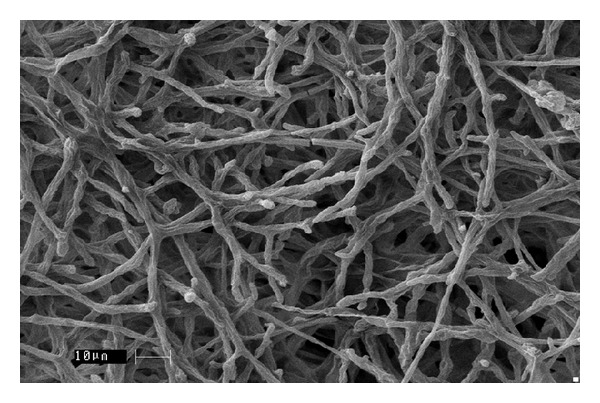
Mycelial hyphae of* M. alpina *CFR-GV15 under SEM image.

**Figure 6:**
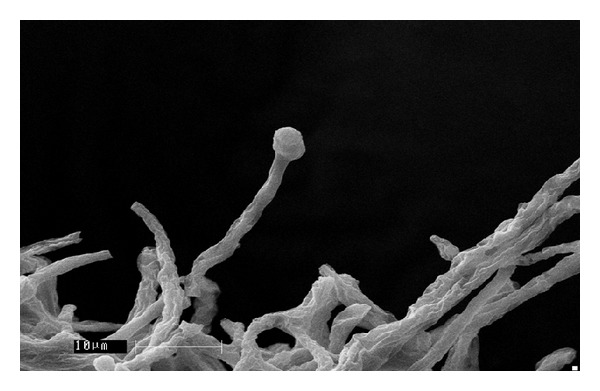
Sporangiospores of* M. alpina* CFR-GV15 under SEM image.

Different growth temperatures play an important role in the formation of roselle petal growth pattern on the growth agar media. Initially a loop of inoculums was placed in the middle of agar culture plate and incubated at 19°C for 5–7 days. While colonial growth was observed on PDA plate, later on these plates were incubated at 28°C for another 3-4 days. Vigorous growth was observed on PDA plate and roselle petal clearly formed in the plate (Figures 1 and 2). More carbon and low nitrogen content in the medium play a vital role in the formation of roselle petal in the PDA plate. All* Mortierella *sp. were saprophytic and proteolytic in nature, and they grew faster on the nutrient-rich medium compared to the other fungi,* Mucor *sp. and* Pythium* sp. Milky white with cotton colonies with pure white or milky growth, in morphological structure similar to* Mortierella* sp. (standard culture) were tentatively identified as per the fungi manual and were taken further for 18S rRNA sequence studies. Septate coenocytic mycelium, swollen sporangiophores, and morphological characters similar to* Mortierella* sp. were observed in our study (Figures 5 and 6). All the morphological characters were additionally compared with standard culture and the Gilman manual [17]. Based on the above observations, 11 species of* Mortierella* were selected and named as CFR-GV11 to CFR-GV19. In these selective isolates* M*.* alpina* CFR-GV15 was screened and stained with TTC to measure the relation between staining degree and lipid content, especially with omega-6 and omega-3 fatty acid. The results were presented in Table 2. Staining degree was found maximum in* M. alpina* CFR-GV15 observed as 1.541 at 458 nm. Total lipid in mycelial structure and higher AA increased during the course of production time. The reduction of TTC [10, 20] is mostly used as a biochemical marker for the viability of living cells. It is generally thought that TTC is reduced by dehydrogenases and it is absorbed by living cells [20], where it reacts with hydrogen atoms released by the dehydrogenase enzymes during cellular respiration [21]. Although there are many dehydrogenases in fungi, only AA-producing fungus* M. alpina* could be stained red. Similarly positive strain of* M. alpina* CFR-GV15 observed maximum amount as 1.541 at 458 nm spectrophotometrically.* Mucor* which is not producing AA could not be stained red, and this indicated that the enzyme dehydrogenase is specific for* Mortierella *sp. and not for* Mucor*. The fatty acid profiles of all 11 selective isolates of* Mortierella *species and two* M. alpina *MTCC and CBS standard strains are shown (Table 3).* M. alpina* CFR-GV15, the total fatty acid content significantly increased maximum of 53.86% AA, 4.87% EPA, and 3.94% DHA whereas the standard culture MTCC 6344 accumulated less amount of total fatty acid content 38.69% AA, 2.65% EPA, and 2.45% DHA. Similarly* M. alpina* CFR-GV15,* M. alpina* CFR-GVM15, and* M. horticola* CFR-GV10 had a higher content of AA, EPA, and DHA. However,* M. elongata* CFR-GV16 and CFR-GV17 and* M. exigus* CFR-GV11 had low yield. A few of our isolates had very negligible amount of these fatty acids. Therefore,* M. alpina* CFR-GV15 was chosen for further study.

### 3.1. Molecular Identification and Phylogenic Tree Prediction of* Mortierella *sp.

**Figure 7:**
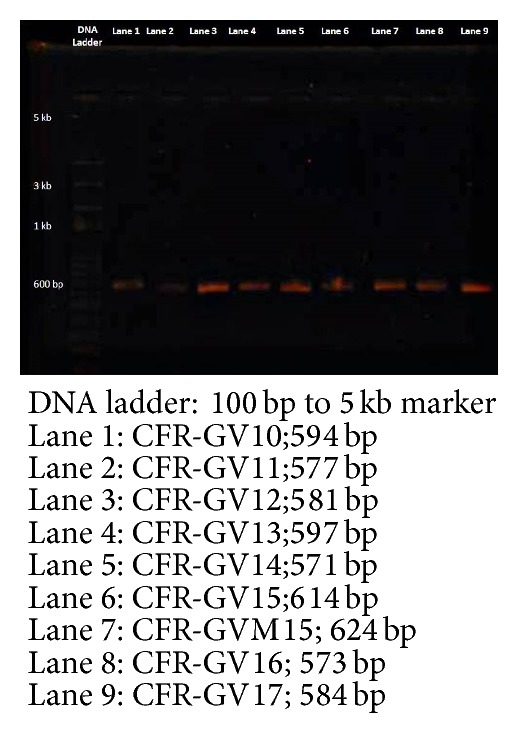
18S rRNA, ITS1 and ITS4 primer, and selective PCR amplified products.

**Figure 8:**
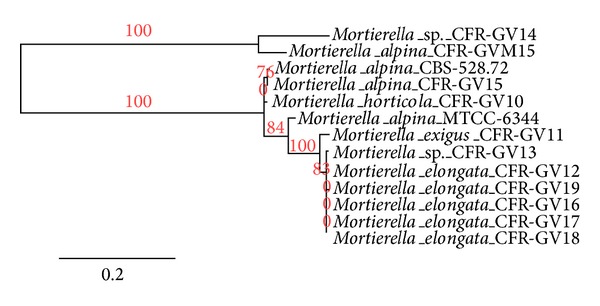
Neighbor-joining phylogenetic analyses of putative isolated PUFAs producing* Mortierella *sp.

The 11 selective isolates of* Mortierella* sp. were sequenced and sent to NCBI GenBank. Accession numbers have been shown in Table 2. A total of 37 isolated DNA were amplified with 18S-28S ribosomal gene internal transcribed spacer (ITS1-rDNA-ITS4 DNA) regions of* Mortierella *sp. from three different locations (Western Ghats of Tamil Nadu, Kerala, and Karnataka, India), sequenced, and investigated. Out of the 11 selective* Mortierella* sp., three closely related species (*M. alpina*,* M. horticola,* and* M. exigus*) showed the most similar sequence identity, were isolated from the Western Ghats of Karnataka soil sample compared to the Western Ghats of Tamil Nadu, and were found to have the highest nucleotide diversities. Omega-6 and omega-3 fatty acid production by different strains of* Mortierella* sp. (*M. alpina, Mortierella elongata*,* Mortierella horticola,* and* Mortierella exigus*) were detected. One of the isolates,* Mortierella alpina *CFR-GV15, was found to be producing the highest amount of omega-6 and omega-3 fatty acids (AA, EPA, and DHA). Molecular characterization of* M. alpina* CFR-GV15 was carried out for taxonomic identifications using 18S rRNA gene sequences. The genomic DNA extracted from* M. alpina *CFR-GV15 by a modified method was found to be of good quality (Figure 7). The rRNA gene amplicon obtained by PCR was 614 bps as expected (Figure 7) and was subjected to sequencing. The gene sequence of the PCR product obtained represents part of the 18S rRNA, ITS1, 5.8S rRNA, ITS4, and 28S rRNA regions. On BLAST analysis,* M. alpina* CFR-GV15 exhibited 76% homology with* M. alpina* CBS 528.72 (accession number AJ271629).* M. alpina* CFR-GV15 was further confirmed as a new strain of* M. alpina* through molecular phylogenetic analysis. It was subsequently subjected to DNA sequencing and BLAST analysis. The phylogenetic tree of ITS amplified sequence generated by phylogenic.org [15] showed that* M. alpina* CFR-GV15 was clustered with* Mortierella* sp. (Figure 8).

### 3.2. Effect of Temperature and Enhanced Production of Omega-3 Fatty Acids

The cultivation temperature is one of the most important environmental factors affecting the growth of microorganisms and causing changes in many biosynthetic pathways. In the present study, profuse growth of* M. alpina* CFR-GV15 was observed at a temperature ranging from 12°C to 28°C with enhanced lipid production (Table 4). The cultivation temperature had a statistically significant difference and was selected based on temperature enhancing the omega-3 fatty acid production in the strain investigated. A significant difference (*P* ≤ 0.05) in higher amount of total biomass 8.41 g/L, total lipid 38.43%, and ARA, EPA, and DHA 30.54%, 5.90%, 4.50%, respectively, obtained at 12°C, whereas significant increase of biomass (11.05 g/L), total lipid (42%), EPA (6.79%), and DHA (4.09%) were obtained first time at 20°C for 4 days and 12°C in 5 days. A study indicated that, at a lower temperature, the fungus produced more eicosapentaenoic acid [18, 19].* M. alpina* 1S-4 produces EPA (approximately 10% of total fatty acids) below the growth temperature of 20°C through n-3 fatty acid pathway and direct *ω*-3 desaturation of AA [22]. The strain produced levels of EPA of more than 0.3 g/L at 12°C. The strain exhibits higher EPA production on the addition of *α*-linolenic acid (18:3n *ω*-3) containing oils, such as linseed oil, to the medium [23]. By using a Δ^12^ desaturase-defective mutant, Mut48, derived from* M. alpina* 1S-4, an EPA-rich oil with a low level of AA was obtained. Like the wild-type strain, Mut48 converted exogenous ALA to EPA [24]. The amount of EPA accumulated reached 1.88 g/L (66.6 mg/g dry mycelia) on cultivation of* M. alpina* 1S-4 with 3% linseed oil at 12°C [25] and, in another report, basal medium was 2.0% of soluble starch and 1.0% of linseed oil as carbon source at initial pH 6.0 and incubated at 20°C for 7 days [26], with the yield of EPA 3.56% and DHA 0.18%. This result can be attributed to the proposed adaptive role of PUFAs in membrane stabilization under stress conditions of low temperatures. PUFAs including ARA, EPA, and DHA may play a vital role in the regulation of membrane fluidity in this organism, thereby compensating for the decreased functionality of the biomembranes under cold stress conditions. The regulation of fatty acid saturation by desaturase enzymes is known as homeoviscous adaptation, wherein the organism adjusts the membrane fluidity to maintain the optimal function of biological membranes. Another possible explanation is that, at a lower temperature, more dissolved oxygen is available in the culture medium for desaturase enzymes that are oxygen dependent [27], thereby resulting in production of more unsaturated fatty acids.

### 3.3. Production and Enhancement of Fatty Acid Content of* M. alpina* CFR-GV15 at 7- and 9-Day Cultivation

**Figure 9:**
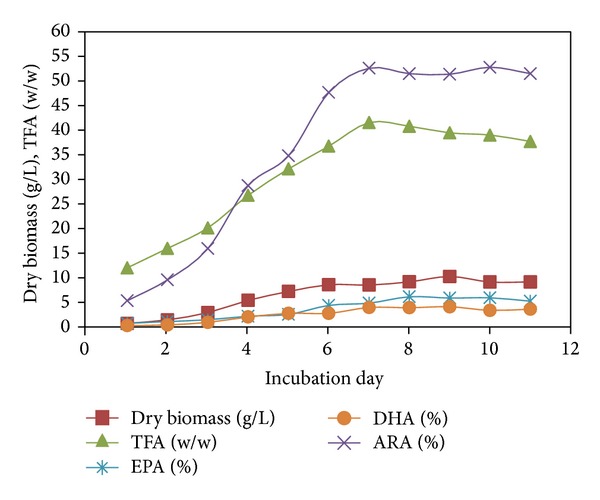
Growth curve of* M. alpina* CFR-GV15 in starch-yeast extract medium.

The changes of fatty acid content of* M. alpina* CFR-GV15 in 7-day cultivation are shown in Figure 9. From days 1 to 11, in contrast, the content of AA in cell dry weight increased from 5.2% to 53.25% from days 1 to 7. High contents of OA and AA further implicated that the starch and potassium nitrate (of which 25% is OA and 58% is AA) [28] were absorbed by* M. alpina* CFR-GV15 at the beginning of cultivation. In addition, the correlation coefficients between contents of C18:1 and AA suggest that there were enzymatic conversions of OA and LA to AA in the fungus* M. alpina* CFR-GV15 during fermentation. In the* M. alpina* PUFA enzymatic process, OA is first converted to LA by Δ12 desaturations, which is then converted to GLA (C18:3, n-6) by *ω*-6 desaturation. Two carbon atoms are then added to the GLA by *ω*-6 specific elongation to form DGLA (C20:3, n-6) which is ultimately converted to AA by *ω*-5 desaturation [8]. The PUFAs content of DGLA, AA, EPA, and DHA in cell dry weight, the significant differences (*P* ≤ 0.05) increased from days 4 to 7 (from 2.75% to 3.78%, from 29% to 53%, from 2.4% to 4.7%, and from 1.84% to 3.8%, resp.). In the stress condition, 2% starch and 0.5% yeast extract and the temperature at 12°C 4 days and 20°C 5 day with the cold adaptation stress condition* M. alpina* CFR-GV15 and further activation of Δ^17^ desaturase converted DGLA to ETA and AA to EPA and further DHA accumulation (data not shown). Previous studies [29, 30] showed that arachidonic acid (Ara) fungi belonging to genus* Mortierella* accumulate EPA in their mycelia when grown in the usual media containing glucose as the major carbon source at low temperature of 6°C–20°C. This phenomenon is probably due to activation of methyl-end-directed desaturation Δ^17^-desaturation of Ara, formed through n-6 fatty acid route, to EPA at low temperature [31]. Particularly at 20°C temperature, AA accumulations are stimulated in* Mortierella* [8]. In lower temperature of 5°C, a temperature-sensitive Δ^17^ desaturase is activated which catalyses the formation of eicosapentaenoic acid [20:5 (*ω*-3)] from 20:4 (*ω*-6) [32]. All significant PUFAs producers (LA, GLA, DGLA, AA, EPA, and DHA) are valuable fatty acids and are beneficial to human health, suggesting that the PUFA-rich oil produced by* M. alpina* CFR-GV15 would have high potential in food and therapeutical application. Together with the amount of fatty acid unsaturation changes, these findings suggested that the fatty acid compositions were tightly regulated in the fungus during different phases of cultivation.

## 4. Conclusion

In conclusion, 37 strains and 11 selective native isolates of* Mortierella* sp. were isolated from soil of the Western Ghats of India and were shown to be a potential PUFA producer including a higher amount of AA producers. In the cultivation media, temperature had a statistically significant difference in biomass and omega-3 fatty acid production in the selective strain, and the significantly highest PUFA-containing strain in this study was identified as* M. alpina* CFR-GV15, which was also confirmed as a new strain of* Mortierella* through the ITS region. It is suitable for distinguishing* Mortierella* species identification and other closely related species for comparison.* M. alpina* CFR-GV15 produced the highest levels of biomass and lipid content at stationary phase (day 7 and day 9), whereas fatty acid contents were continuously changed during cultivation. This study would also contribute to the improvement and supplement of some additional nutrient for the PUFA production and add to our understanding of fatty acid metabolism in this fungus* M. alpina*. Nowadays, renewable sources of EPA from algae, yeast, fungi, and other microorganisms are available. Microorganism-based processes seem to be reliable and economically attractive source of omega-3 fatty acid especially EPA and DHA and can provide an efficient way for large-scale production.

## Figures and Tables

**Table 1:** Collection of saprophytic soil samples from the different species of *Mortierella* growing in the Western Ghats regions of Tamil Nadu, Kerala, and Karnataka. The total cultivable fungi and the total specific species of *Mortierella* (state-wise).

S1 number	Place of collection	Number of soil samples	Total number of fungi	Total *Mortierella* species
1	Mysore			
	Bandipur wild forest	02	20	0

2	Tamil Nadu			
	Mudumalai wild forest	20	15	02
	Nilambur-Gudalur road	12	20	08

3	Kerala			
	Nilambur road	04	15	04
	Vazhikkadavu	03	12	04
	Karimpuzha bridge	03	08	02
	Payyavoor road	02	11	0
	Chemperi road	02	16	0

4	Karnataka			
	Sullia	03	15	02
	Puttur	03	14	03
	Ujere	04	10	03
	Dharmasthala	02	5	0
	Subramanya	01	14	0
	W.G*1-W.G7	16	34	1
	W.G8	03	35	0
	W.G10	03	25	02
	W.G11	03	26	02
	W.G12	03	38	05
	W.G13	04	34	0
	Sakleshpur	04	12	0

	Total	67	379	37

W.G*: Western Ghats.

**Table 2:** Biomass production and lipid content of 11 isolates of *Mortierella  *sp. after 7 days of cultivation.

Fungal isolates	Strains identified	Genebank accession number	Staining degree (A485 nm)	Dry biomass (g/L)	Total lipid content (%)
MV1	*Mortierella horticola *CFR-GV10	KF743701	0.812 ± 0.66^e^	8.52 ± 0.30^c^	34.60 ± 5.10^c^
M08	*Mortierella exigus* CFR-GV11	KF743702	0.761 ± 0.00^c^	8.24 ± 2.30^c^	39.00 ± 3.60^ab^
2aS2	*Mortierella elongata* CFR-GV12	KF743703	0.528 ± 0.04^h^	7.85 ± 0.01^d^	38.70 ± 0.20^b^
M01	*Mortierella *sp. CFR-GV13	KF798172	0.706 ± 0.03^f^	5.83 ± 4.05 ^g^	41.02 ± 0.80^a^
S2	*Mortierella *sp. CFR-GV14	KF798173	0.936 ± 0.35^c^	6.37 ± 0.60^f^	38.89 ± 0.70^b^
**M05**	*Mortierella alpina* CFR-GV15	**KF561137 **	1.541 ± 0.02^**a**^	10.82 ± 1.9^**a**^	39.05 ± 0.40^**ab**^
M06	*Mortierella alpina* CFR-GVM15	KF683921	1.099 ± 0.04^b^	7.85 ± 0.80^d^	40.00 ± 2.30^a^
M02	*Mortierella elongata* CFR-GV16	KF561138	0.641 ± 0.21^g^	8.42 ± 3.01^c^	37.90 ± 0.54^bc^
M27	*Mortierella elongata* CFR-GV17	KF568908	0.683 ± 0.25^g^	9.02 ± 1.10^b^	28.98 ± 0.40^e^
M07	*Mortierella elongata* CFR-GV18	KF679986	0.941 ± 0.45^c^	8.45 ± 0.20^c^	35.62 ± 0.12^c^
M21	*Mortierella elongata* CFR-GV19	KF679987	0.329 ± 0.14^i^	7.42 ± 4.02^de^	38.36 ± 0.71^bc^
MA1	*Mortierella alpina* MTCC 6344	KC018186	0.688 ± 0.58^g^	4.30 ± 0.20^i^	29.07 ± 0.50^e^
MA2	*Mortierella alpina* CBS 528.72	AJ271629	0.845 ± 0.41^d^	6.52 ± 5.08^f^	37.42 ± 0.20^bc^

Culture conditions: starch-yeast extract medium; pH: 6.5 and incubated at 20°C; carbon source: 2% starch, 0.5% yeast extract; 1% KNO_3_; 0.1% KH_2_PO_4_ and MgSO_4_·7H_2_O 0.05%; 230 rpm; cultivation period: 7-day values of means ± SD, n-3. Values in the same column that do not share the same alphabetic superscripts are significantly different at *P* ≤ 0.05 levels according to Duncan's multiple range test.

**Table 3:** Total fatty acid profile of newly isolated *Mortierella *sp.

Strain	C14:0	C16:1	C18:0	C18:1	C18:2	C18:3	C20:3	C20:4	C20:5	C22:6
CFR-GV11	0.96 ± 0.2^d^	23.14 ± 0.2^ab^	5.67 ± 0.8^fg^	29.78 ± 0.1^b^	7.17 ± 0.2^a^	3.86 ± 0.3^d^	3.00 ± 0.8^d^	32.03 ± 0.0^f^	1.34 ± 0.8^g^	1.56 ± 0.2^bcd^
CFR-GV12	0.62 ± 0.4^f^	21.02 ± 0.1^d^	4.56 ± 0.1^i^	32.02 ± 0.8^a^	6.45 ± 0.1^bcd^	4.62 ± 0.2^c^	4.20 ± 0.5^a^	32.54 ± 0.2^f^	3.65 ± 0.2^b^	3.48 ± 0.5^ab^
CFR-GV13	0.68 ± 0.1^f^	18.52 ± 0.3^e^	4.98 ± 0.4^i^	24.52 ± 0.1^e^	5.23 ± 0.4^ef^	3.26 ± 0.0^f^	1.98 ± 0.6^h^	28.32 ± 0.6^g^	3.12 ± 0.1^c^	2.36 ± 0.1^abcd^
CFR-GV14	0.86 ± 0.2^e^	16.35 ± 0.2^f^	5.68 ± 0.3^fg^	23.16 ± 0.7^f^	5.68 ± 0.3^e^	3.35 ± 0.4^ef^	2.45 ± 0.1^ef^	27.56 ± 0.4^h^	3.26 ± 0.6^c^	1.78 ± 0.3^bcd^
CFR-GV15	0.48 ± 0.0^h^	10.66 ± 0.4^i^	5.05 ± 0.3^h^	6.21 ± 0.5^i^	6.07 ± 0.5^d^	5.58 ± 0.2^a^	3.37 ± 0.3^c^	53.86 ± 0.2^a^	4.87 ± 0.0^a^	3.94 ± 0.6^b^
CFR-GVM15	0.54 ± 0.7^g^	9.85 ± 0.2^i^	4.58 ± 0.7^i^	6.00 ± 0.1^i^	5.21 ± 0.2^e^	5.24 ± 0.6^b^	2.75 ± 0.6^e^	48.25 ± 0.1^b^	2.35 ± 0.6^de^	3.68 ± 0.2^ab^
CFR-GV16	1.92 ± 0.3^c^	20.87 ± 0.8^d^	5.58 ± 0.1^f^	29.58 ± 0.4^c^	6.37 ± 0.1^bcd^	4.58 ± 0.5^c^	1.89 ± 0.7^h^	26.11 ± 0.3^i^	1.91 ± 0.4^f^	0.68 ± 0.8^d^
CFR-GV17	2.30 ± 0.4^b^	22.81 ± 0.1^c^	7.11 ± 0.5^cd^	30.27 ± 0.1^b^	6.15 ± 0.6^d^	3.15 ± 0.3^f^	2.56 ± 0.6^ef^	22.03 ± 0.9^i^	2.02 ± 0.3^ef^	0.65 ± 0.6^d^
CFR-GV18	1.89 ± 0.2^c^	22.01 ± 0.9^c^	6.89 ± 0.9^e^	27.52 ± 0.6^d^	4.25 ± 0.4^g^	2.87 ± 0.4^g^	2.15 ± 0.1^g^	37.12 ± 0.7^d^	0.78 ± 0.0^h^	0.68 ± 0.5^d^
CFR-GV19	2.03 ± 0.1^a^	23.75 ± 0.0^a^	7.50 ± 0.8^c^	30.35 ± 0.8^b^	6.33 ± 0.2^cd^	3.46 ± 0.1^de^	2.77 ± 0.6^e^	22.01 ± 0.6^k^	1.31 ± 0.3^g^	0.88 ± 0.3^cd^
MTCC 6344	0.45 ± 0.2^h^	14.25 ± 0.4^g^	11.25 ± 0.1^a^	24.00 ± 0.9^e^	6.90 ± 0.8^b^	1.05 ± 0.1^h^	3.90 ± 0.4^b^	38.69 ± 0.5^c^	2.65 ± 0.1^d^	2.45 ± 0.2^abcd^
CBS528.72	0.57 ± 0.4^g^	13.01 ± 0.2^h^	10.65 ± 0.6^b^	18.53 ± 0.4^g^	6.61 ± 0.9^bc^	0.84 ± 0.2^i^	3.70 ± 0.0^b^	34.86 ± 0.1^e^	3.29 ± 0.5^bc^	4.04 ± 0.0^a^

Culture conditions: starch-yeast extract medium; pH: 6.5 and incubated at 20°C; carbon source: 2% starch, 0.5% yeast extract; 1% KNO_3_; 0.1% KH_2_PO_4_ and MgSO_4_·7H_2_O 0.05%; 230 rpm; cultivation period: 7-day values of means ± SD, n-3. Values in the same column that do not share the same alphabetic superscripts are significantly different at *P* ≤ 0.05 levels according to Duncan's multiple range test.

**Table 4:** Effect of temperature on enhancement of biomass, total lipid, and EPA and DHA production by *M.alpina  *CFR-GV15.

Temperature	Dry biomass g/L	Total lipid % w/w	ARA content %	EPA content %	DHA content %
20°C for 4 days and 12°C for 5 days*	11.82 ± 0.21^a^	42.01 ± 0.25^b^	23.56 ± 0.81^e^	6.79 ± 0.41^a^	4.09 ± 0.40^b^
12°C	8.41 ± 0.10^d^	38.34 ± 0.11^d^	30.54 ± 0.52^d^	5.90 ± 0.01^b^	4.50 ± 0.56^a^
15°C	9.80 ± 0.25^c^	40.20 ± 0.25^c^	47.20 ± 0.85^c^	4.21 ± 0.49^d^	4.20 ± 0.72^a^
20°C	10.45 ± 0.52^b^	41.89 ± 0.52^b^	53.70 ± 0.07^b^	4.80 ± 0.14^c^	3.92 ± 0.45^c^
28°C	10.86 ± 0.22^b^	44.25 ± 0.78^a^	56.82 ± 0.24^a^	3.46 ± 0.75^d^	4.30 ± 0.90^a^

Culture conditions: starch-yeast extract medium; pH: 6.5; carbon source: 2% starch, 0.5% yeast extract; 1% KNO_3_; 0.1% KH_2_PO_4_ and MgSO_4_·7H_2_O 0.05%; 230 rpm; cultivation period: 7-day values of means ± SD, n-3. *Cultivation period: 9-day values of means ± SD, n-3. Values in the same column that do not share the same alphabetic superscripts are significantly different at *P* ≤ 0.05 levels according to Duncan's multiple range tests.
